# The functions and mechanisms of prefoldin complex and prefoldin-subunits

**DOI:** 10.1186/s13578-020-00446-8

**Published:** 2020-07-20

**Authors:** Jiaxin Liang, Longzheng Xia, Linda Oyang, Jinguan Lin, Shiming Tan, Pin Yi, Yaqian Han, Xia Luo, Hui Wang, Lu Tang, Qing Pan, Yutong Tian, Shan Rao, Min Su, Yingrui Shi, Deliang Cao, Yujuan Zhou, Qianjin Liao

**Affiliations:** 1 Hunan Key Laboratory of Translational Radiation Oncology, Hunan Cancer Hospital and the Affiliated Cancer Hospital of Xiangya School of Medicine, Central South University, 283 Tongzipo Road, Changsha, 410013 Hunan China; 2 Department of Medical Microbiology Immunology & Cell Biology, Simmons Cancer Institute, Southern Illinois University School of Medicine, 913 N. Rutledge Street, Springfield, IL 62794 USA

**Keywords:** Prefoldin complex, Prefoldin subunits, Protein folding, Prefoldin-like complex, Neurodegenerative diseases, MM-1, c-Myc

## Abstract

The correct folding is a key process for a protein to acquire its functional structure and conformation. Prefoldin is a well-known chaperone protein that regulates the correct folding of proteins. Prefoldin plays a crucial role in the pathogenesis of common neurodegenerative diseases (Alzheimer’s disease, Parkinson’s disease, and Huntington’s disease). The important role of prefoldin in emerging fields (such as nanoparticles, biomaterials) and tumors has attracted widespread attention. Also, each of the prefoldin subunits has different and independent functions from the prefoldin complex. It has abnormal expression in different tumors and plays an important role in tumorigenesis and development, especially c-Myc binding protein MM-1. MM-1 can inhibit the activity of c-Myc through various mechanisms to regulate tumor growth. Therefore, an in-depth analysis of the complex functions of prefoldin and their subunits is helpful to understand the mechanisms of protein misfolding and the pathogenesis of diseases caused by misfolded aggregation.

## Background

Prefoldin complex was discovered 20 years ago in the domains of eukaryotes and archaea as the property of promoting the assembly of cytoskeletal proteins (actin and tubulin) into corresponding polymers, also known as Gim complex (Genes involved in microtubule biogenesis) [1]. The prefoldin complex helps protein fold correctly and prevents aggregation by providing class II chaperones (Hsp60 molecular chaperones found in archaebacteria and eukaryotic cytoplasm) with a linear, unnatural substrate in the cytoplasm [2]. Therefore, the Prefoldin complex plays a role as a chaperone protein [3].

As a cytoplasmic chaperone protein, the prefoldin complex is a hybrid oligomer assembled from six different proteins (six subunits). The prefoldin complex is a multifunctional protein, and its importance has been known. Studies have shown that one subunit of prefoldin affects the protein levels of other subunits [4]. By forming complexes with other constituent subunits, the prefoldin subunit is protected from degradation mediated by the ubiquitin–proteasome system [4]. However, it is unclear how cells regulate the protein level of each subunit, and what is the mechanism to regulate the activity ratio between subunits and their complexes. Studies have found that the absence of specific prefoldin subunits, rather than the prefoldin complex in Saccharomyces cerevisiae will alter stress-induced transcription [5]. Therefore, we have reason to believe that each subunit also has different and independent functions from the complex, and each prefoldin subunit confers different substrate specificity to the prefoldin complex [6]. In various tumors, the subunit of prefoldin was abnormally expressed. Therefore, it is necessary to understand the role of prefoldin subunits in the development of different diseases (such as tumors).

Therefore, the importance of in-depth analysis of the complex functions of the prefoldin complex and its subunits is self-evident. In this article, we update the latest developments in the functions and mechanisms of the prefoldin complex and prefoldin subunit.

## Prefoldin complex

### Structure of the Prefoldin complex

**Fig. 1:**
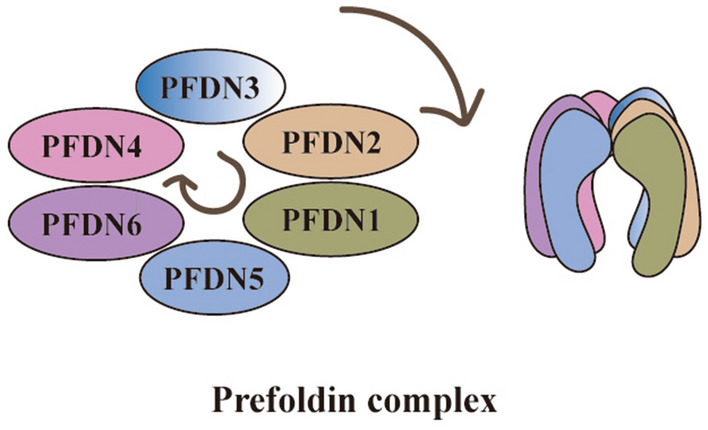
Schematic of Prefoldin complex. Canonical prefoldin complex is a heterohexameric complex composed of two α subunits (PFDN3 and PFDN5) and four β subunits (PFDN1, PFDN2, PFDN4 and PFDN6)

Archaebacteria prefoldin complex is composed of two identical α subunits and four identical β subunits, forming an α2β4 hexamer (Fig. 1) [7]. Eukaryotic prefoldin complex is a heterohexameric complex like a jellyfish-like structure, consisting of six different subunits (PFDN1–6): two α subunits (PFDN3 and PFDN5) and four β subunits (PFDN1, PFDN2, PFDN4, and PFDN6) [8]. From the perspective of spatial structure, “jellyfish” is composed of double beta barrels and six slender tentacle-shaped coils that have hydrophobic amino acid residues at the distal end to bind unnatural proteins [9]. Regardless of archaea or eukaryotes, each alpha subunit terminal junction region of the prefoldin complex contains two beta hairpins, and each beta subunit contains one beta-hairpin [10]. Chaperones, such as prefoldin, triggering factor and Hsp90 all have a clamp-like structure that holds the substrate proteins. The clamp-like structure has a multivalent binding surface and can protect the protein conformers of unnatural proteins until they reach Natural state or transfer to another component of the folding machine [11].The heterohexameric complex is first composed of two sub-complexes: PFDN2–PFDN3 and PFDN5–PFDN6, and finally assembled by PFDN1 and PFDN4. The prefoldin subunits are arranged in the clockwise space order of PFDN3–PFDN2–PFDN1–PFDN5–PFDN6–PFDN4.

### Function of the prefoldin complex

#### Prefoldin complex acts as chaperone protein in cytoplasm

Both actin and tubulin are important components of the eukaryotic cytoskeleton. Due to their high concentration, they are easily self-binding and cannot be folded correctly. Therefore, newly synthesized actin and tubulin require an assistant, and the eukaryotic prefoldin complex can establish the correct tubular assembly for many tubular proteins (such as actin, α/β tubulin). The prefoldin complex specifically binds to the cytoplasmic chaperone protein of TCP-1 (CCT), a loop complex, forming actin molecules and acting as a transport molecule that directs protein accurately, thereby promoting actin and tubulin to be protected from aggregation and folded correctly [12]. Researchers have observed in yeast that in the presence of the prefoldin complex, the CCT folding rate can be increased five times, which may be related to the prefoldin complex that prevents the premature release of newborn proteins [13].

**Fig. 2:**
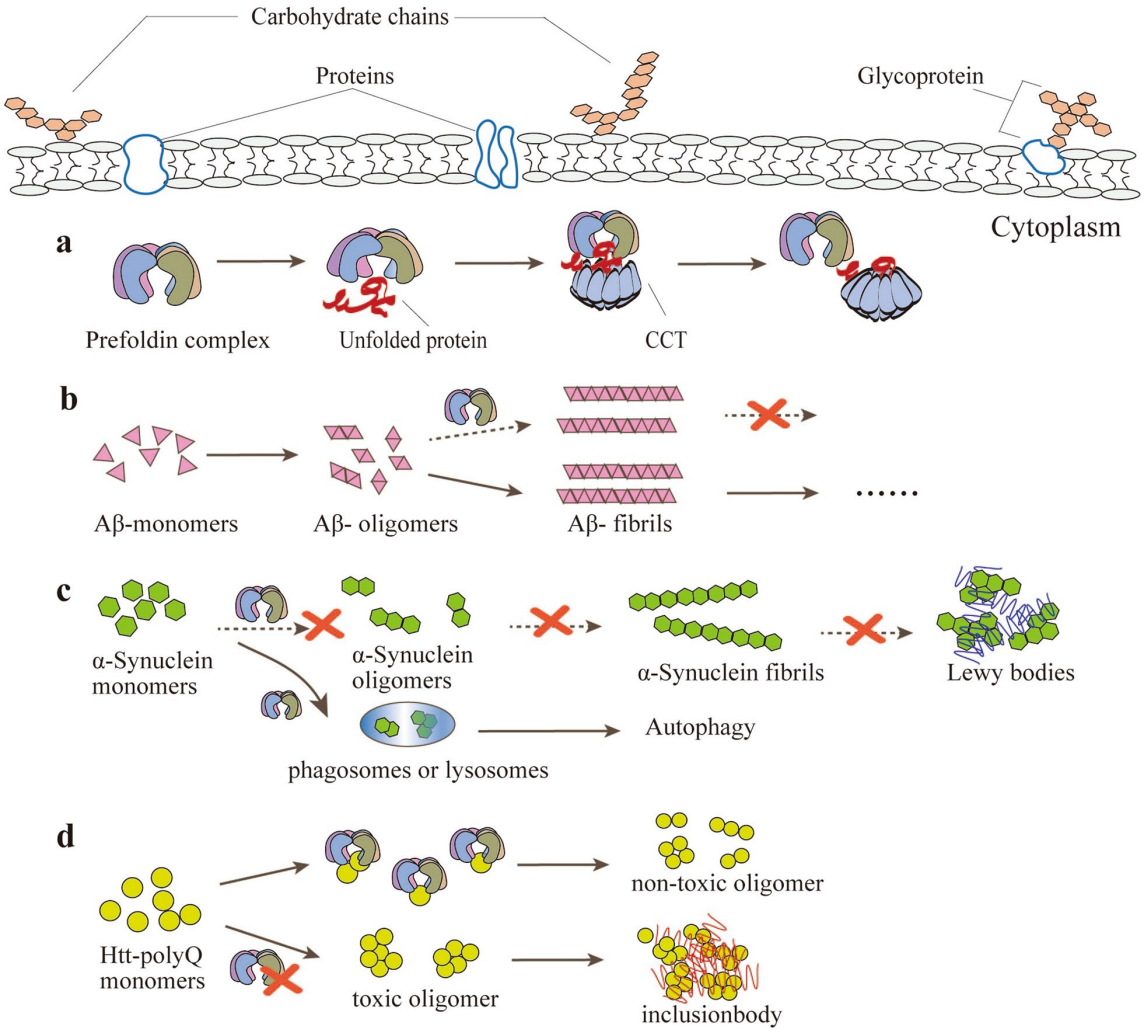
Function of the Prefoldin Complex. **a** Prefoldin complex acts as a chaperone protein in the cytoplasm. Prefoldin specifically binds to the cytoplasmic chaperone protein of the loop complex containing TCP-1 (CCT) and acts as a transport molecule to direct the target protein. Once the unfolded protein transported by the prefoldin complex contacts the CCT, Prefoldin will be released automatically and leave the active site. **b** Schematic model of prefoldin complex against pathogenic forms of Alzheimer’s disease. Human prefoldin (hPFD) can inhibit Aβ fibril formation and contribute to the non-toxic aggregation of Aβ. **c** The prefoldin complex prevents the pathogenesis of Parkinson’s disease. The prefoldin complex inhibits the early stages of α-synuclein aggregation and assists in autophagy-dependent degradation of α-synuclein-causing mutants by delivering them to lysosomes. **d** Schematic model of prefoldin complex against pathogenic forms of Huntingtin. Prefoldin complexes can help Huntington proteins to form non-toxic oligomers (dimers to tetramers), and the lack of prefoldin complexes can produce toxic oligomer and inclusion body

It is well known that chaperone proteins are a class of functional proteins that recognize unnatural, immature, and conformationally unstable proteins and help them to fold, assemble, and transport correctly. After assembly, the chaperone protein is actively separated and does not form part of the protein structure that performs the function. Therefore, once tubulin transported by prefoldin complex contacts CCT, the prefoldin complex is automatically released and leaves the active site due to the high affinity of tubulin for CCT (Fig. 2a). When the prefoldin complex is contacted with CCT, it will lose its affinity for the unfolded target protein [14]. Therefore, prefoldin complex binds only to unnatural unfolded target proteins in the cytosol. Unlike many other chaperone proteins, the substrate binding and release of the prefoldin complex are not related to adenosine triphosphate (ATP) [15].

#### Prefoldin complex are involved in the pathogenesis of neurodegenerative diseases

The role of prefoldin complex as a chaperone-mediated folding is not limited to cytoskeleton components, but also involved in the assembly of other cytoplasmic complexes and maintains protein function by avoiding protein aggregation and promoting proteolysis and degradation. Recent studies show that prefoldin complexes can promote proteasome degradation of cytosolic proteins with missense mutations by maintaining substrate solubility, which reflects the role of prefoldin complexes in preventing the accumulation of potentially toxic proteins [16]. Studies have shown that the prefoldin complex is involved in the pathogenesis of neurodegenerative diseases, especially Alzheimer’s disease (AD) [17], Parkinson’s disease (PD) [18] and Huntington’s disease (HD) [19].

Extracellular aggregation of the β-amyloid peptide (Aβ) in the human cerebral cortex and marginal regions is one of the onset features of AD, and the oligomer structure of Aβ is related to its toxicity (Fig. 2b) [20]. The production of Aβ is a complex self-assembly process. Once abnormality occurs during the assembly process, proteins are abnormally aggregated and misfolded, and Aβ will be abnormally stacked. Chaperone proteins can play a significant role in the breakdown of protein aggregates, and thus chaperone proteins are essential in cell defense (e.g., protein aggregation caused by misfolding inside and outside the cell) and are also potentially powerful neurodegeneration inhibitors [21]. Previous studies have shown that the expression of chaperone proteins Hsp60, Hsp70, and Hsp90 is enhanced or down-regulated in AD-affected tissues and cells, indicating that Hsp60, Hsp70, and Hsp90 are lively in the development and progression of AD disease [21]. Karin, et al. found that human prefoldin (hPFD) can inhibit the formation of Aβ fibrils and help the non-toxic aggregation of Aβ, indicating that prefoldin complex may have a protective effect on AD [17]. Parkinson’s disease (PD), also known as tremor paralysis, is a long-term degenerative disease of the central nervous system that mainly affects the motor system [22]. Alpha-synuclein is a pathogenic gene product of familial PD and is the main component of inclusion bodies in PD [23]. Studies have shown that the prefoldin complex not only inhibits the early stages of α-synuclein aggregation but also assists in autophagy-dependent degradation of α-synuclein-causing mutants by delivering them to lysosomes (Fig. 2c) [18]. Huntington’s disease (HD), also known as hereditary chorea, is an autosomal dominant hereditary neurodegenerative disease. The main cause of HD is the mutation of the Huntington gene (IT15) on the patient’s chromosome 4 leading to the synthesis of the misfolded form of the toxic Huntington protein (mHTT) [24]. Abnormal Huntington protein has many repeated glutamines and is easy to stick and aggregate, eventually leading to the death of nerve cells [25]. Prefoldin complex retains Huntington protein oligomers at the small oligomer stage (dimer to tetramer), thereby inhibiting the aggregation and extension of larger pathogenic Huntington protein oligomers and the formation of inclusion bodies (Fig. 2d) [19, 26].

Besides, the prefoldin complex plays a key role in maintaining neuronal cell activity. Studies have shown that the prefoldin complex activity and the ability to prevent protein aggregation are stronger in neuronal cells than non-neuronal cells: PFDN1-deficient mice show cerebellar neuron loss [27]; missense mutations in PFDN5 can cause neurodegeneration [6]. In Drosophila, the prefoldin complex and infertile partners (Pins) synergistically regulate the asymmetric division of neuroblasts and intermediate neural progenitor cells (INPs) by stabilizing tubulin, and hence inhibits neuroblast overgrowth in the brain of Drosophila larvae [28].

Although neurodegenerative diseases are currently incurable and treatment aims to improve symptoms, with the in-depth study of the role and mechanism of the prefoldin complex in neurodegenerative diseases, it is expected to develop drugs for the treatment of neurodegenerative diseases that target prefoldin.

#### Application of prefoldin complex in emerging fields

The prefoldin complex does not only play an important role in neurodegenerative diseases but also in emerging fields. Djohan et al. found that the prefoldin complex exists on the surface of gold nanoparticles (AuNPs), and the presence of the prefoldin complex helps to synthesize AuNPs with dispersion stability and particle controllability [29]. Organic solvents are extremely toxic to bacteria, and the accumulation of organic solvents can damage microbial cell membranes, thereby affecting cell structure and functional integrity [30]. Studies have found that *E. coli* cells that highly express prefoldin and class II chaperone proteins are resistant to organic solvents. This is because prefoldin prevents the accumulation of organic solvents in *E. coli* cells by preventing the activity of intramolecular chaperones [31]. Samuel et al. found that from hyperthermophilic archaea γ-prefoldin (γPFD) functions as a protein component in a novel protein-polymer hybrid hydrogel. The resulting hybrid hydrogel has adjustable mechanical properties and can be used as the design of biological materials (such as tissue culture scaffolds and wound adhesives), as well as multi-step biocatalysis and stem cell culture [32]. γ-prefoldin does not form oligomeric oligomers with α-prefoldin or β-prefoldin but rather forms filaments of a certain size so that it is also known as filamentous protein γ-prefoldin [33]. Nanotechnology is attractive because it uses proteins as templates to locate molecules in a regular pattern with nano-precision, which is necessary to build advanced biomaterials. The filamentous protein γPFD, through incremental gene truncation, has achieved controllable attachment of filaments in a specific direction on the carbon surface to form oriented filaments, so that it is expected to be used as a biological template [34].

## The multiple roles of prefoldin subunits

**Fig. 3:**
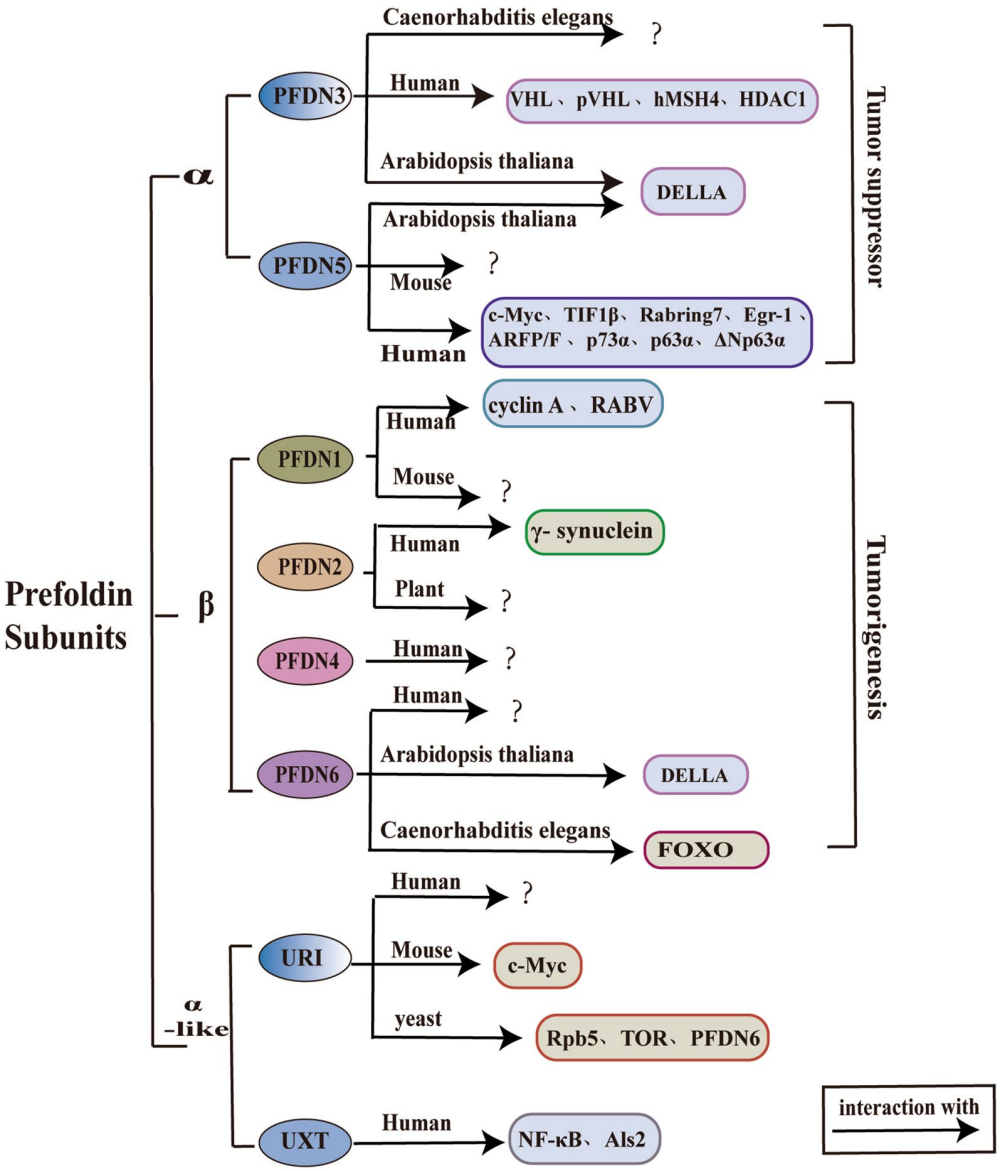
Multiple roles and physical interactions with proteins of prefoldin subunits. Prefoldin subunits can play an important role in different species, but the specific molecular interaction mechanism has not been elucidated yet (denoted by “?” in the figure). The molecules in the boxes indicated by the arrows can interact with the prefoldin subunits. The alpha subunits (PFDN3 and PFDN5) of prefoldin play a tumor-suppressing role, and the beta subunits (PFDN1, PFDN2, PFDN4, and PFDN6) play a role in tumorigenesis. VHL: Von Hippel-Lindau; pVHL: von Hippel-Lindau protein; hMSH4: human MutS homologs 4; HDAC1: Histone deacetylase 1; DELLA: integrators of gibberellin (GA) action; TIF1β: transcriptional intermediary factor–1β; Rabring7: Rab7‐interacting ring finger protein; Egr-1: early growth response 1; ARFP/F: ARFP (alternative reading frame protein)/F (for frameshift); p73α and p63α: a member of the p53 family; ΔNp63α: the dominant negative isoform of the p63 family; cyclin A: cell cycle protein A; RABV: Rabies virus; FOXO: forkhead box class O; Rpb5: RNA polymerase II (RNAPII) subunit 5; TOR: target of rapamycin; PFDN6: prefoldin subunit 6; NF-κB; nuclear factor-kappaB; and Als2: amyotrophic lateral sclerosis type 2

The prefoldin complex is multifunctional, and its importance is self-evident. It has been found that prefoldin subunits can play different roles in different species and diseases (such as tumors) (Fig. 3), and the abnormal expression of prefoldin subunits occurs in many tumors. Thus it is necessary to have an in-depth understanding of the functions and roles of the prefoldin subunits.

### The role of prefoldin subunits in human tumors and other diseases

The study found that the prefoldin subunit can be used as a strong indicator of poor prognosis of gastric cancer (GC), and the results of subgroup analysis in various clinical parameters show that different prefoldin subunits display different effects the overall survival situation (Table 1) [35].

**Table 1:** Proteins that physically interact with canonical or prefoldin-like subunits, and research advances in different organisms and diseases

Prefoldin subunits	Function	Organism	Interactor	Disease
β				
PFDN1	Oncogene	Human	Cyclin A [36] RABV [39]	Gastric cancer [35] Lung cancer [36] Colon cancer [37] Nasopharyngeal carcinoma [38]
		Mouse		Lymphocyte development [27] Cytoskeletal defect [27]
PFDN2	Oncogene	Human	γ-Synuclein [42]	Gastric cancer [35] Breast cancer [40] Metastatic urothelial carcinoma [41]
		Plant [65]		
PFDN4	Oncogene	Human		Atherosclerosis [19] Gastric cancer [35, 52] Breast cancer [48, 49] Colon cancer [51] Hepatocellular carcinoma [50] Atopic dermatitis [53]
PFDN6		Human		Gastric cancer [35] Acute lymphocytic leukemia [64]
		*Arabidopsis thaliana* [76]	DELLA [67]	
		*Caenorhabditis elegans*	FOXO [77]	
α				
PFDN3/VBP1	Anti-oncogene	Human	VHL [43] pVHL [44] hMSH4 [45] HDAC1 [46]	Gastric cancer [35]
		*Arabidopsis thaliana*	DELLA [67]	
		*Caenorhabditis elegans* [66]		
PFDN5/MM1	Anti-oncogene	Human	c-Myc [99] TIF1β [101] Rabring7 [127] Egr-1 [128] ARFP/F [129] p73α [133] p63α [134] ΔNp63α [135]	Gastric cancer [35] Secondary hyperparathyroidism [54, 55] Colon cancer [60]
		Mouse		Breast cancer [70– 73]
		*Arabidopsis thaliana*	DELLA [67]	
α-like				
URI/Bud27		Human		Cervical cancer [89] Colon cancer [90] Gastric cancer [91]
		Mouse	c-Myc [88]	Hepatocellular carcinoma [85]
		Yeast	Rpb5 [83] TOR [93] PFDN6 [94]	
UXT/Art-27			NF-κB [95] Als2 [97]	Androgen receptor [96] Centrosome component [98]
γ				
γ-prefoldin				Nanobiotechnology [32–34]

PFDN1 has been reported to be involved in the development of many types of tumors (such as lung, breast, and colon cancers) [36, 37]. PFDN1 is a cancer-promoting factor. In lung cancer, PFDN1 inhibits the expression of cyclin A by directly interacting with the cyclin A promoter at the transcription initiation site, thereby suppressing EMT and metastasis of lung cancer [36]; In colon cancer cell lines SW480 and RKO cells, silencing PFDN1 can inhibit the proliferation, invasion, and migration of colon cancer cells, and PFDN1 expression is positively correlated with tumor size and tumor invasion, therefore PFDN1 can be used as an indicator of poor prognosis of colorectal cancer [37]. In other disease, there is no lack of research on PFDN1. Through the reference gene selection system for nasopharyngeal carcinoma gene expression research, PFDN1 was found to be one of the candidate genes [38]. Studies have found that in N2a cells infected with rabies virus (RABV), the subcellular distribution of PFDN1 has been changed and redistributed to the characteristic Negri-body-like (NBL) structure in the cytoplasm [39].

PFDN2 plays an oncogene role in glioblastoma, breast, pancreatic, and colon cancers [40]. Most breast cancer (BC) shows resistance to taxanes due to changes in tubulin genes, and PFDN2 is one of the neighboring proteins that can interact with sexual tubulin [40]. In two cohort studies of metastatic urothelial carcinoma, an increase in 1q23.3 copy number was found to be associated with low survival, and PFDN2 is one of the genes located in 1q23.3, which is closely related to poor prognosis [41]. A common hallmark of glaucoma is the loss of retinal ganglion cells (RGCs), while γ-synuclein (SNCG) is highly expressed in somatic cells and synapses in RGCs, and PFDN2 can be a candidate upstream regulator to identify SNCG expression [42]. Other studies have shown that the depletion of PFDN2 leads to the formation of ectopic neuroblasts [28].

PFDN3, also known as Von Hippel-Lindau binding protein 1 (VBP1), is a chaperone protein that binds to VHL. VBP1 interacts with the tumor suppressor VHL. VHL acts as a chaperone protein, which changes the intracellular localization of VBP1 from cytoplasm to the nucleus [43]. VBP1 may be a tumor suppressor protein. VBP1 interacts with pVHL (an E3 ubiquitin ligase) in vitro and enhances its stability, which degrades HIF-1α in an oxygen-dependent manner and inhibits epithelial–mesenchymal transition (EMT) caused by HIF-1α [44]. Studies have shown that VBP1 and VHL can also interact with p97, a AAA (+) ATPase involved in protein degradation and DNA damage, thereby affecting the polyubiquitination of the human MutS protein family hMSH4 [45]. VBP1 is co-localized with nuclear HDAC1, indicating that the delivery of HDAC1 to the CCT complex occurred in the nucleus [46].

PFDN4, also known as C-1, can function as a transcription factor or cofactor in cell cycle regulation [47]. The expression of PFDN4 may be closely related to the occurrence and development of various tumors (such as breast cancer [48, 49], hepatocellular carcinoma [50], and colorectal cancer [51]) and poor prognosis. In TWIST-depleted gastric cancer cells, PFDN4 expression is up-regulated, and EMT-related morphological and molecular changes promoted by TWIST can be reversed [52]. In addition, accumulation of low-density lipoprotein cholesterol (LDL-c) in the arterial wall is closely related to the initiation and progression of atherosclerosis, and PFDN4 has biological functions related to LDL [19]. A genome-wide association study of Japanese-specific dermatitis just identified PFDN4 as one of eight new susceptible loci [53].

PFDN5 is differentially expressed in thyroid tumor tissue [54], and abnormal expression of PFDN5 that associated with protein synthesis and processing has been detected in secondary hyperparathyroidism [55]. Uveitis is the most common extra-articular manifestation of ankylosing spondylitis (AS). Compared with AS patients without uveitis, AS patients with uveitis had significantly higher serum PFDN5 levels, and PFDN5 had a protective effect on uveitis cell death, suggesting that PFDN5 can be used as a biomarker for AS uveitis [56]. Knocking down the candidate transcriptional regulator PFDN5 can induce the differentiation of human embryonic stem cells (hESC) [57]. In memory CD4+ T cells activated in asthma [58] and the immature CD4+ T lymphocytes in systemic lupus erythematosus [59], the mRNA levels of differential genes were detected by reverse transcription analysis, and elevated PFDN5 mRNA levels were detected. In analysis of differential gene expression profiles of colon cancer, it was identified that PFDN5 is a possible marker for the clinical prognosis of colorectal cancer [60]. In addition, PFDN5 is closely related to the pathogenesis of neurodegenerative diseases [61].

PFDN6 is a functionally unknown hydrophilic protein (KE2) in a major histocompatibility complex [62]. Response to dexamethasone (DEXA) is one of the key prognostic factors for predicting the outcome of acute lymphocytic leukemia (ALL) [63]. Studies on dexamethasone-resistant leukemia cells have found that PFDN6 may play a potential biomarker role in the prognosis and chemotherapy of ALL [64].

### The role of the prefoldin subunits in other species

Studies have shown that PFDN1-deficient mice exhibit phenotypes of cytoskeletal functional defects. For instance, ciliary dyskinesia, B cells, and T cells are significantly damaged in the early stages of maturation, suggesting that PFDN1 is necessary for lymphocyte development and function [27]. Difficult destructuring and saccharification of plant cell walls is one of the main obstacles to the development of lignocellulosic materials, and targeting genes involved in cell wall biosynthesis can reduce recalcitrant. PFDN2 is one of the genes involved in cell wall biosynthesis and can affect the permeability of plant cell walls, but the specific mechanism is not clear [65]. PFDN3/VBP1 has homologs in mice, *Drosophila* and *C. elegans* [66]. Effective chaperone-mediated tubulin biogenesis is crucial in *C. elegans*. In the absence of PFDN3, *C. elegans* displays gonad developmental defects including abnormal distal tip cell migration [66]. DELLA protein is diurnally regulated by gibberellin (GA) plant hormones and interacts with prefoldin subunits, especially PFDN3 and PFDN5, providing a possible mechanism for cortical microtubule formation [67].

Recent studies have identified a link between hypercholesterolemia and cognitive deficits. Studies have shown that PFDN5 may be an important component of synaptic plasticity in the hippocampus of mice [68] and exposed to hypercholesterolemia in New Zealand white rabbits’ prefrontal cortex (PFC), PFDN5 is one of the genes whose expression is down-regulated [69]. Therefore, the study of the PFDN5 gene may help to clarify the mechanism of the link between hypercholesterolemia and cognitive deficits. Like women, breast tumors are the most common tumors in female dogs. Multi-branched DNA (b-DNA) analysis of canine breast tumor frozen specimens revealed that PFDN5 expression was significantly lower in malignant tumors [70, 71]. PFDN5 gene somatic cell deletion is more common in canine breast cancer and it is common and closely related to high Ki-67 scores [72, 73], indicating that down-regulation or deletion of PFDN5 expression has a very important role in the development of canine breast cancer. Also, it was observed that PFDN5nmf5a mutant mice are susceptible to syndromes characterized by photoreceptor degeneration, central nervous system abnormalities, and male infertility, which may be related to the reduction of microtubule and microfilament formation caused by missense mutations in PFDN5 [6]. L110R mice with PFDN5 missense mutations have defective spermatogenesis and reduced expression of sperm-related genes, which are manifested as male infertility [74]. Bob1 (a homolog of PFDN5) can regulate sex differentiation in yeast by interacting with the MAP kinase BYR1 [75].

In plant-related studies, nuclear accumulation of PFDN5 and PFDN6 occurs in wild-type Arabidopsis in a DELLA-dependent manner, suggesting that accumulation of DELLA protein is necessary for the localization of these two subunits in the nucleus [67]. It is also observed in Arabidopsis that the PFDN6-1 mutant can cause microtubule and cell division defects [76]. In Caenorhabditis elegans, heat shock factor 1 (HSF-1) can increase the expression of PFDN6, promote the binding of PFDN6 to the long-lived gene FOXO, and enhance the transcriptional activity of FOXO, thereby extending the lifespan of *C. elegans* [77].

### Prefoldin subunits are involved in the assemble of prefoldin-like complex

**Fig. 4:**
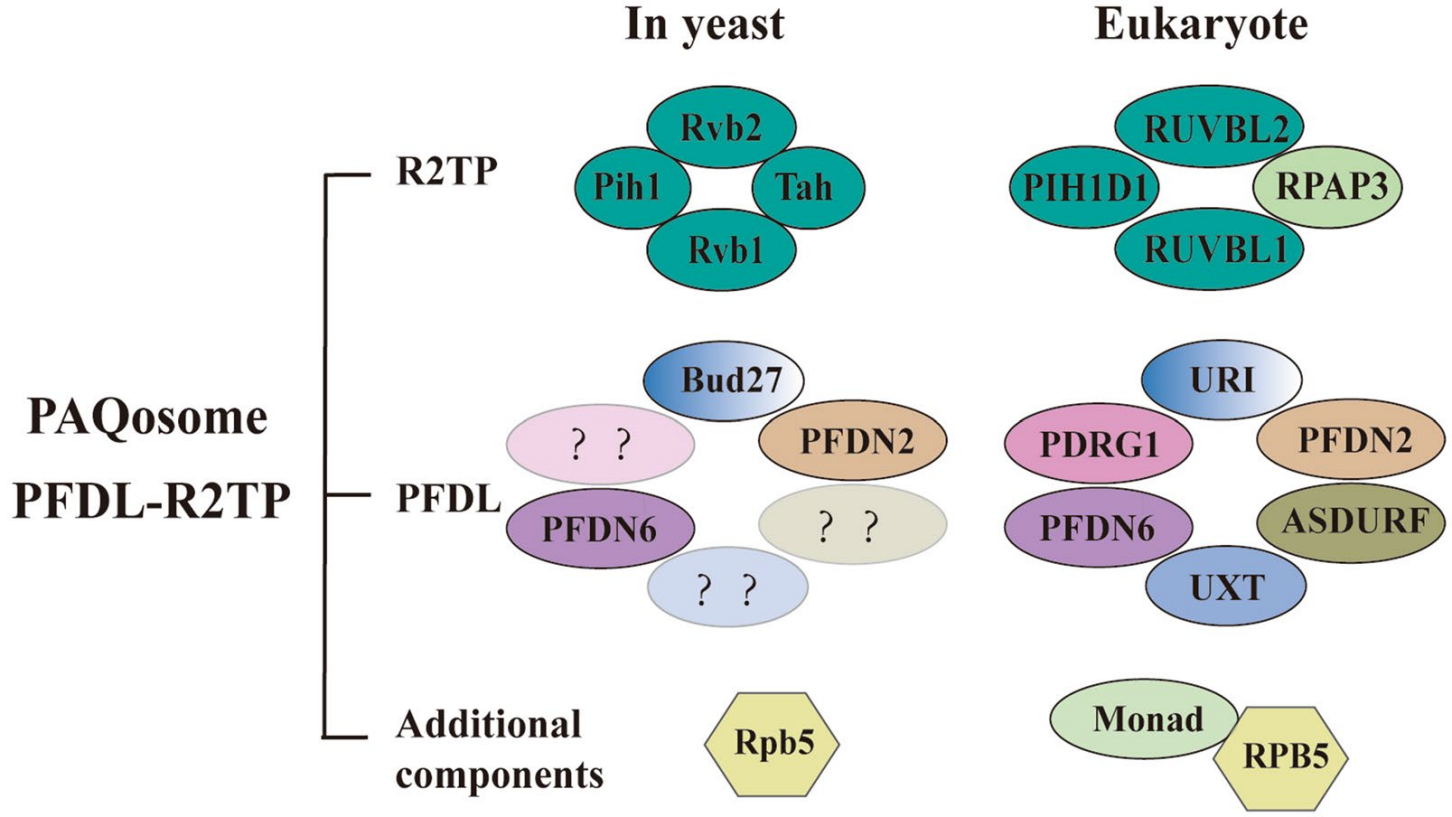
Schematic of PAQosome (PFDL-R2TP). In addition to canonical prefoldin complexes, eukaryotes also have prefoldin-like complexes (PFDL). Among them, the classic α subunits were replaced with (URI and UXT) and some classic β subunits were replaced with PDRG1 and ASDURF. PFDL interacts with other chaperone proteins, such as R2TP complex (RUVBL1-RUVBL2-RPAP3-PIH1D1) to form PFDL-R2TP (PAQosome). PAQosome also has additional components Monad and RPB5. Corresponding homologs exist in yeast

The classic β subunits of prefoldin PFDN2 and PFDN6 can form a heterohexameric complex with URI/RMP, UXT/Art-27, PDRG1 and ASDURF (Fig. 4) [78]. This is a protein complex with weak sequence homology to the prefoldin complex and thus called the prefoldin-like complex (PFDL) [79]. Unlike the prefoldin complex, PFDL is involved in the cytoplasmic assembly of RNA polymerase II [80]. PFDL can interact with another chaperone protein (like R2TP), to form an R2TP/PFDL complex, also named PAQosome, a 12-subunit chaperone protein [81]. Among them, the subunit compositions of R2TP (RUVBL1-RUVBL2-RPAP3-PIH1D1) is different in yeast and humans [82]. PAQosome also contains two attachments, Monad/WDR92 and RPB5/POLR2E (Fig. 4) [83]. PAQosome can assist in the assembly of HSP90 and is involved many basic cellular functions (e.g., protein synthesis, ribosome biogenesis, transcription, splicing, etc.) [81]; it also helps to stabilize and assemble phosphatidylinositol-3 kinase-related protein kinases [84].

Similarly, each subunit of the prefoldin-like complex also functions independently of the complex. URI is the alpha subunit of PFDL and is also called RNA polymerase II fifth subunit regulatory protein (RMP) [85]. In the cytoplasm, URI acts as a chaperone protein; in the nucleus, URI acts as a transcription regulator and can interact with RPB5 (a subunit common to three eukaryotic RNA polymerases) and RNA polymerase II (pol II) [86, 87]. URI has strong oncogenic activity because it can regulate the functions of many proteins including transcription factors (ERα and AhR) [86]. For example, in a mouse model, excessive expression of URI in hepatocytes can lead to hepatocellular carcinoma (HCC) [85]; decreasing the expression of URI in the intestine will activate the c-Myc expression induced by β-catenin, leading to mouse cell proliferation, DNA damage, and susceptibility to fatal gastrointestinal syndrome (GIS) caused by ionizing radiation (IR) [88]. In addition, URI can induce resistance of cervical cancer [89], colorectal cancer [90], and gastric cancer cells [91], and promote cell survival. Bud27, the URI yeast homolog, participates in the cytoplasmic assembly of three nuclear RNA polymerases in an Rpb5-dependent manner, and therefore plays an important role in transcription extension [92]. Bud27 can also mediate gene expression controlled by TOR, and the TOR pathway is involved in processes involving ribosome biogenesis [93]. Studies have also shown that Bud27 only physically interacts with the PFDN6 component in R2TP/PFDL [94].

Another alpha subunit of PFDL, UXT/Art-27, is a cofactor. Studies have shown that UXT can form a dynamic complex with NF-κB and is recruited to enhancers of NF-κB after stimulation, as an important part of NF-κB transcription enhancer [95]. Studies have shown that this subunit can interact with the N-terminus of the androgen receptor (AR) and can play a role in promoting receptor-induced transcriptional activation [96]. It has been reported that UXT can interact with Als2, a gene that causes mutations in autosomal recessive forms of motor neuron disease, and the interaction of Als2 and UXT has an important role in the activation of the NF-κB pathway [97]. Also, UXT is a component of centrosome and is associated with γ-tubulin, which is essential for cell viability [98].

## Prefoldin subunit 5 (mm-1) and c-myc

### MM-1 Isoforms

The PFDN5 subunit is the most well-known of the six subunits of prefoldin. PFDN5 is also called MM-1 (Myc Modulator-1), a protein that can interact with Myc [99, 100]. MM-1 has four isoforms, namely MM-1α, MM-1β, MM-1γ, and MM-1δ. The four isoforms of MM-1 have different cellular localizations with c-Myc and have different degrees of inhibitory activity on c-Myc [101]. Isoform MM-1α is the main expression form of MM-1 in cells. MM-1β is universally expressed in tissues (except the heart and small intestine), while MM-1γ and MM-1δ are strongly expressed in fetal tissues. MM-1β and MM-1δ are mainly localized in the cytoplasm, while MM-1α and MM-1γ are localized in the nucleus, and MM1 isoforms that bind to c-Myc and TIF1β are located in the nucleus [101].

### MM-1, a c-Myc binding protein

**Fig. 5:**
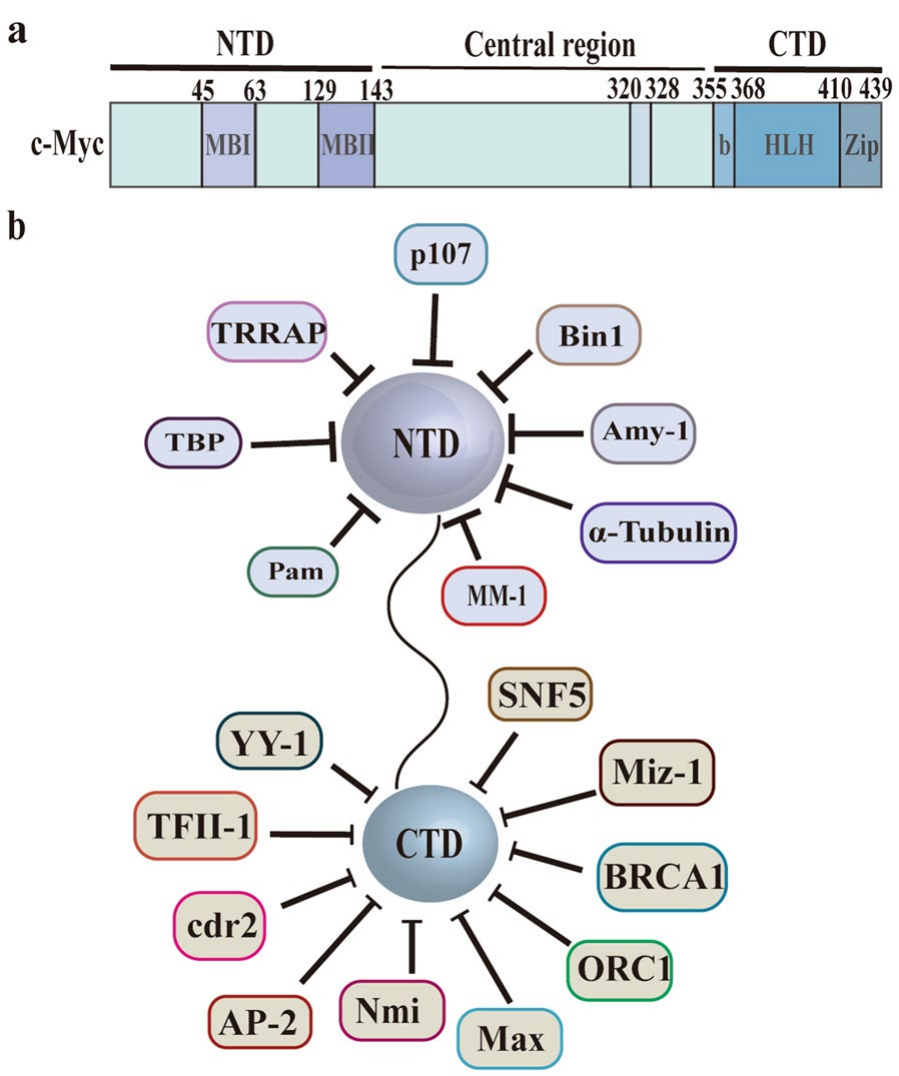
Functional domains of human c-Myc protein. **a** The human c-Myc protein has two main functional domains: the carboxy-terminal domain (CTD) and the amino-terminal domain (NTD). CTD harbors the basic (b) helix–loop–helix (HLH) leucine zipper (LZ). NTD harbors conserved ‘MYC Boxes’ I and II (MBI and MBII). **b** CTD-interacting proteins: AP2, BRCA1, cdr2, CBF-C, CDC6, Miz-1, MSSPs, Nmi, ORC1, SNF5 and YY-1; NTD-interacting proteins: α-Tubulin, AMY-1, BIN-1, p21, p107, PAM, TBP, and TRRAP. MM-1 is one of the c-Myc NTD-interacting proteins which is bound to the MBII region

Abnormal expression of the oncogene c-Myc often results in 30–50% of human malignancies. C-Myc not only participates in regulating a variety of cellular metabolic pathways (such as glucose metabolism, glutamine metabolism, and serine metabolism), but also plays a core role as a transcription factor in life activities, such as cell cycle regulation, cell differentiation, and protein synthesis and aging [102]. C-Myc has two main functional domains (Fig. 5a): the carboxy-terminal domain (CTD) and the amino-terminal domain (NTD) [103]. CTD has a basic (b) helix-loop-helix (HLH) leucine zipper (LZ) motif. This region can dimerize with Max and physiologically recognize the DNA target sequence, which is required for all biological behaviors [104]. NTD can participate in transcriptional regulation. It has two short segments of “Myc box” I and II (MBI and MBII) which are conserved in all Myc family proteins and trans-activates the c-Myc target genes [105].

The c-Myc binding proteins can be roughly divided into two groups: the protein that binds b-HLH-Zip at the CTD end and the protein that binds MBI or MBII at the NTD end. c-Myc binding proteins positively or negatively regulate the tumorigenicity and transcriptional activity of c-Myc. The proteins that interact with c-Myc CTD are (Fig. 5b): AP2 [106], BRCA1 [107], cdr2 [108], CBF-C(NF-YC) [109] ,CDC6 [110], Miz-1 [111], MSSPs [112], Nmi [107], ORC1 [113], SNF5 [114] and YY-1 [115, 116]; NTD-binding proteins are (Fig. 5b): α-Tubulin [117], AMY-1 [118], BIN-1 [119], p21 (cip1/waf1/sdi1) [120], p107 [121, 122], PAM [123], TBP [124] and TRRAP [125]. MM-1 is one of the c-Myc NTD-terminated proteins which is bound to the MBII region of the NTD and can compete with TRRAP for c-Myc [99].

### The regulatory mechanisms of MM-1 on c-Myc

**Fig. 6:**
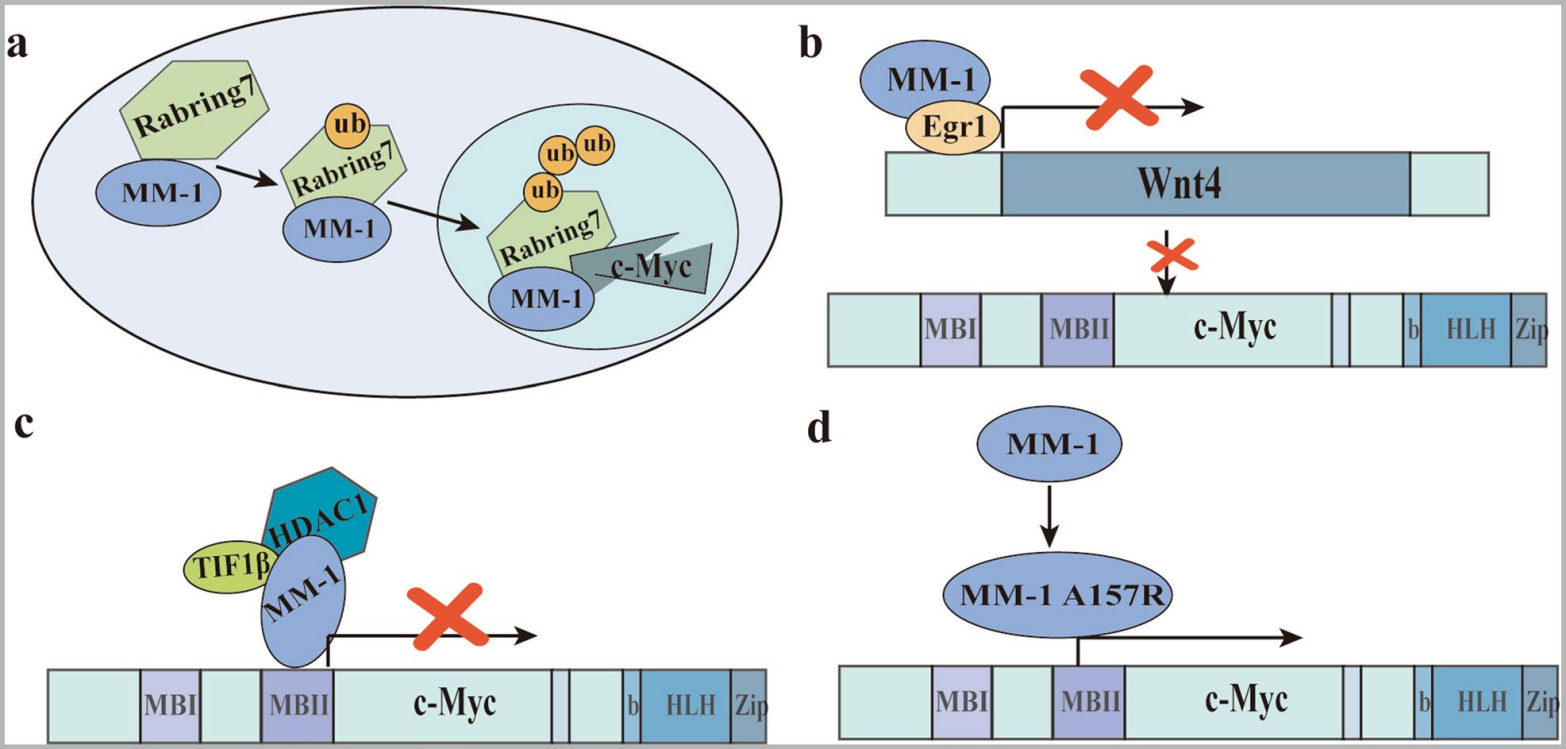
Four regulatory mechanisms of MM-1 on c-Myc. **a** Rabring7 degrades c-Myc through complex formation with MM-1. **b** MM-1 and Egr1 cooperate in the transcriptional repression of Wnt4, which is one of the elements of the Wnt-b-catenin pathway that positively controls the c-Myc gene. **c** MM-1 binds the N-terminal region of c-Myc, and represses its transcriptional activity by recruiting the TIF1β co-repressor and the histone deacetylase HDAC1–mSin3 complex. **d** Inhibition of c-Myc activity by MM-1 can be lost due to the missense mutation of amino acid 157 from alanine to arginine (A157R)

MM1, a nuclear c-Myc binding protein, inhibits c-Myc activity in the nucleus in various ways and is therefore considered a tumor suppressor. As showed in Fig. 5, the regulatory mechanisms of c-Myc mediated by MM-1 are: (a) MM-1 promotes c-Myc degradation by recruiting proteasomes and a novel ubiquitin E3 ligase [126]. For example, MM-1 forms a complex with Rabring7 (a protein containing RING fingers and binding to Rab7) to degrade c-Myc (Fig. 6a) [127]. (b) MM-1 regulates the classic Wnt-β-catenin pathway. MM-1 negatively regulates the expression of wnt4 by binding to Egr-1, thereby indirectly inhibiting c-Myc expression (Fig. 6b) [128]. (c)MM-1 inhibits the E-box-dependent transcriptional activity of c-Myc by recruiting a histone deacetylase (HDAC-mSin3) complex from TIF1β/KAP1/TRIM28 (a transcriptional co-inhibitor) (Fig. 6c) [99]. For example, the ARFP/F protein of the hepatitis C virus (HCV) can enhance the transactivation activity of the c-Myc gene by antagonizing the inhibitory effect of MM-1 [129]. Among them, the oncogene c-FMS acts as a target gene involved in the c-Myc-MM-1-TIF1β pathway, and its expression and promoter activity are up-regulated [130]. (d) Inhibition of c-Myc activity by MM-1 can be lost due to the missense mutation of amino acid 157 from alanine to arginine (A157R) (Fig. 6d). A157R mutants are reported to be frequently observed in approximately 50–60% of patients with leukemia or lymphoma [131].

Both p63 and p73 are paralogs with high sequence homology to p53, all three are tumor suppressors, and each has a region in the sequence that is responsible for identifying and binding the target gene sequence, which is called DNA binding domain (DBD). This area is prone to aggregate and form amyloid fibers [132]. It has been reported that MM-1 can interact with p73α rather than p73β, and the expression of MM-1 greatly reduces c-Myc-mediated inhibitory activity on p73α [133]. p63α regulates the activity of c-Myc by directly interacting and regulating the stability of MM-1 protein, which leads to cell cycle progression and tumorigenesis [134]. ΔNp63α mediates the ubiquitination of c-Myc modulator MM-1 through the E3 ligase HERC3 to regulate cell senescence and tumorigenesis [135]. Therefore, to explore the regulatory effect of MM-1 on c-Myc, the key is the upstream molecules of MM-1.

## Conclusion

Proper folding of proteins is a key process for a protein to acquire its functional structure and conformation. As an important chaperone protein, prefoldin is involved in the correct folding of proteins, especially the correct folding of tubulin and actin. Prefoldin plays an important role in the development and progression of neurodegenerative diseases. Advanced research on prefoldin may help to develop drugs for treating neurodegenerative diseases. Besides, abnormal expressions of prefoldin subunits occur in different tumors, and play an important role in the occurrence and development of tumors. Prefoldin subunits are expected to serve as molecular targets for tumor prognosis and drug development. Therefore, an advanced understanding of the complex functions of prefoldin can help to analyze the mechanisms of protein misfolding and the pathogenesis of diseases caused by misfolding aggregation.

However, there are still many unresolved problems in prefoldin: 1. The chaperone protein Hsp90 not only has the function of promoting protein folding but also has the function of promoting the release of exosomes. Then, what about prefoldin? 2. Are related changes caused by deletion and mutation of prefoldin in plants and animals only occurring in specific species? 3. Is there a prominent subunit in the six subunits of prefoldin? 4. Is the prefoldin complex stable? When does the independent function of the Prefoldin subunit appear, before or after the formation of the Prefoldin complex? 5. Is the stability of the complex changed due to some factors? No matter what, the functions of the prefoldin complex and the unique functions of the prefoldin subunit need to be further explored.

## Data Availability

Not applicable.

## Abbreviations

ATP: Adenosine triphosphate
AD: Alzheimer’s disease
PD: Parkinson’s disease
HD: Huntington’s disease
Aβ: β-Amyloid peptide
hPFD: Human prefoldin
Pins: Infertile partners
INPs: Intermediate neural progenitor cells
AuNPs: Gold nanoparticles
γPFD: γ-Prefoldin
GC: Gastric cancer
RABV: Rabies virus
NBL: Negri-body-like
BC: Breast cancer
RGCs: Retinal ganglion cells
SNCG: γ-Synuclein
VBP1: Von Hippel-Lindau binding protein 1
EMT: Epithelial–mesenchymal transition
LDL-c: Low-density lipoprotein cholesterol
AS: Ankylosing spondylitis
hESC: Human embryonic stem cells
DEXA: Dexamethasone
ALL: Acute lymphocytic leukemia
GA: Gibberellin
PFC: Prefrontal cortex
b-DNA: Branched DNA
HSF-1: Heat shock factor 1
PFDL: Prefoldin-like complex
pol II: RNA polymerase II
HCC: Hepatocellular carcinoma
GIS: Gastrointestinal syndrome
IR: Ionizing radiation
AR: Androgen receptor
MM-1: Myc modulator-1
HLH: Helix-loop-helix
LZ: Leucine zipper
HCV: Hepatitis C virus
CTD: Carboxy-terminal domain
NTD: Amino-terminal domain
DBD: DNA binding domain

## References

[1] Geissler S, Siegers K, Schiebel E (1998). A novel protein complex promoting formation of functional alpha- and gamma-tubulin. EMBO J.

[2] Arranz R, Martin-Benito J, Valpuesta JM (2018). Structure and function of the cochaperone prefoldin. Adv Exp Med Biol.

[3] Lim S, Glover DJ, Clark DS (2018). Prefoldins in archaea. Adv Exp Med Biol.

[4] Miyazawa M, Tashiro E, Kitaura H, Maita H, Suto H, Iguchi-Ariga SM (2011). Prefoldin subunits are protected from ubiquitin-proteasome system-mediated degradation by forming complex with other constituent subunits. J Biol Chem.

[5] Amorim AF, Pinto D, Kuras L, Fernandes L (2017). Absence of Gim proteins, but not GimC complex, alters stress-induced transcription. Biochim Biophys Acta Gene Regul Mech..

[6] Lee Y, Smith RS, Jordan W, King BL, Won J, Valpuesta JM (2011). Prefoldin 5 is required for normal sensory and neuronal development in a murine model. J Biol Chem.

[7] Leroux MR, Fandrich M, Klunker D, Siegers K, Lupas AN, Brown JR (1999). MtGimC, a novel archaeal chaperone related to the eukaryotic chaperonin cofactor GimC/prefoldin. EMBO J.

[8] Sahlan M, Zako T, Yohda M (2018). Prefoldin, a jellyfish-like molecular chaperone: functional cooperation with a group II chaperonin and beyond. Biophys Rev..

[9] Martin-Benito J, Gomez-Reino J, Stirling PC, Lundin VF, Gomez-Puertas P, Boskovic J (2007). Divergent substrate-binding mechanisms reveal an evolutionary specialization of eukaryotic prefoldin compared to its archaeal counterpart. Structure..

[10] Siegert R, Leroux MR, Scheufler C, Hartl FU, Moarefi I (2000). Structure of the molecular chaperone prefoldin: unique interaction of multiple coiled coil tentacles with unfolded proteins. Cell.

[11] Stirling PC, Bakhoum SF, Feigl AB, Leroux MR (2006). Convergent evolution of clamp-like binding sites in diverse chaperones. Nat Struct Mol Biol.

[12] Lundin VF, Leroux MR, Stirling PC (2010). Quality control of cytoskeletal proteins and human disease. Trends Biochem Sci.

[13] Hartl FU, Hayer-Hartl M (2002). Molecular chaperones in the cytosol: from nascent chain to folded protein. Science.

[14] Zako T, Iizuka R, Okochi M, Nomura T, Ueno T, Tadakuma H (2005). Facilitated release of substrate protein from prefoldin by chaperonin. FEBS Lett.

[15] Hongo K, Itai H, Mizobata T, Kawata Y (2012). Varied effects of *Pyrococcus furiosus* prefoldin and *P. furiosus* chaperonin on the refolding reactions of substrate proteins. J Biochem.

[16] Comyn SA, Young BP, Loewen CJ, Mayor T (2016). Prefoldin promotes proteasomal degradation of cytosolic proteins with missense mutations by maintaining substrate solubility. PLoS Genet.

[17] Sorgjerd KM, Zako T, Sakono M, Stirling PC, Leroux MR, Saito T (2013). Human prefoldin inhibits amyloid-beta (Abeta) fibrillation and contributes to formation of nontoxic Abeta aggregates. Biochemistry.

[18] Takano M, Tashiro E, Kitamura A, Maita H, Iguchi-Ariga SM, Kinjo M (2014). Prefoldin prevents aggregation of alpha-synuclein. Brain Res.

[19] Tashiro E, Zako T, Muto H, Itoo Y, Sorgjerd K, Terada N (2013). Prefoldin protects neuronal cells from polyglutamine toxicity by preventing aggregation formation. J Biol Chem.

[20] van der Kant R, Goldstein LSB, Ossenkoppele R (2020). Amyloid-beta-independent regulators of tau pathology in Alzheimer disease. Nat Rev Neurosci.

[21] Marino Gammazza A, Bavisotto CC, Barone R, de Macario EC, Macario AJ (2016). Alzheimer’s disease and molecular chaperones: current knowledge and the future of chaperonotherapy. Curr Pharm Des.

[22] Raza C, Anjum R, Shakeel NUA (2019). Parkinson’s disease: mechanisms, translational models and management strategies. Life Sci.

[23] Shahnawaz M, Mukherjee A, Pritzkow S, Mendez N, Rabadia P, Liu X (2020). Discriminating alpha-synuclein strains in Parkinson’s disease and multiple system atrophy. Nature.

[24] Kumar A, Kumar V, Singh K, Kumar S, Kim YS, Lee YM (2020). Therapeutic advances for Huntington’s disease. Brain Sci..

[25] Walker FO (2007). Huntington’s disease. Lancet.

[26] Tang B, Seredenina T, Coppola G, Kuhn A, Geschwind DH, Luthi-Carter R (2011). Gene expression profiling of R6/2 transgenic mice with different CAG repeat lengths reveals genes associated with disease onset and progression in Huntington’s disease. Neurobiol Dis.

[27] Cao S, Carlesso G, Osipovich AB, Llanes J, Lin Q, Hoek KL (2008). Subunit 1 of the prefoldin chaperone complex is required for lymphocyte development and function. J Immunol..

[28] Zhang Y, Rai M, Wang C, Gonzalez C, Wang H (2016). Prefoldin and pins synergistically regulate asymmetric division and suppress dedifferentiation. Sci Rep..

[29] Djohan Y, Azukizawa T, Patmawati, Sakai K, Yano Y, Sato F (2019). Molecular chaperone prefoldin-assisted biosynthesis of gold nanoparticles with improved size distribution and dispersion. Biomater Sci..

[30] Topanurak S, Sinchaikul S, Phutrakul S, Sookkheo B, Chen ST (2005). Proteomics viewed on stress response of thermophilic bacterium *Bacillus stearothermophilus* TLS33. Proteomics.

[31] Okochi M, Kanie K, Kurimoto M, Yohda M, Honda H (2008). Overexpression of prefoldin from the hyperthermophilic archaeum *Pyrococcus horikoshii* OT3 endowed *Escherichia coli* with organic solvent tolerance. Appl Microbiol Biotechnol.

[32] Lim S, Jung GA, Muckom RJ, Glover DJ, Clark DS (2019). Engineering bioorthogonal protein-polymer hybrid hydrogel as a functional protein immobilization platform. Chem Commun.

[33] Whitehead TA, Boonyaratanakornkit BB, Hollrigl V, Clark DS (2007). A filamentous molecular chaperone of the prefoldin family from the deep-sea hyperthermophile *Methanocaldococcus jannaschii*. Protein Sci.

[34] Whitehead TA, Je E, Clark DS (2009). Rational shape engineering of the filamentous protein gamma prefoldin through incremental gene truncation. Biopolymers.

[35] Yesseyeva G, Aikemu B, Hong H, Yu C, Dong F, Sun J (2020). Prefoldin subunits (PFDN1-6) serve as poor prognostic markers in gastric cancer. Biosci Rep..

[36] Wang D, Shi W, Tang Y, Liu Y, He K, Hu Y (2017). Prefoldin 1 promotes EMT and lung cancer progression by suppressing cyclin A expression. Oncogene.

[37] Wang P, Zhao J, Yang X, Guan S, Feng H, Han D (2015). PFDN1, an indicator for colorectal cancer prognosis, enhances tumor cell proliferation and motility through cytoskeletal reorganization. Med Oncol.

[38] Guo Y, Chen JX, Yang S, Fu XP, Zhang Z, Chen KH (2010). Selection of reliable reference genes for gene expression study in nasopharyngeal carcinoma. Acta Pharmacol Sin.

[39] Zhang J, Han Q, Song Y, Chen Q, Xia X (2015). Analysis of subcellular prefoldin 1 redistribution during rabies virus infection. Jundishapur J Microbiol..

[40] Nami B, Wang Z (2018). Genetics and expression profile of the tubulin gene superfamily in breast cancer subtypes and its relation to taxane resistance. Cancers..

[41] Riester M, Werner L, Bellmunt J, Selvarajah S, Guancial EA, Weir BA (2014). Integrative analysis of 1q23.3 copy-number gain in metastatic urothelial carcinoma. Clin Cancer Res.

[42] Chintalapudi SR, Jablonski MM (2017). Systems genetics analysis to identify the genetic modulation of a glaucoma-associated gene. Methods Mol Biol.

[43] Tsuchiya H, Iseda T, Hino O (1996). Identification of a novel protein (VBP-1) binding to the von Hippel-Lindau (VHL) tumor suppressor gene product. Cancer Res.

[44] Kim JA, Choi DK, Min JS, Kang I, Kim JC, Kim S (2018). VBP1 represses cancer metastasis by enhancing HIF-1alpha degradation induced by pVHL. FEBS J.

[45] Xu Y, Her C (2013). VBP1 facilitates proteasome and autophagy-mediated degradation of MutS homologue hMSH4. FASEB J..

[46] Banks CAS, Miah S, Adams MK, Eubanks CG, Thornton JL, Florens L (2018). Differential HDAC1/2 network analysis reveals a role for prefoldin/CCT in HDAC1/2 complex assembly. Sci Rep..

[47] Iijima M, Kano Y, Nohno T, Namba M (1996). Cloning of cDNA with possible transcription factor activity at the G1-S phase transition in human fibroblast cell lines. Acta Med Okayama.

[48] Collins C, Volik S, Kowbel D, Ginzinger D, Ylstra B, Cloutier T (2001). Comprehensive genome sequence analysis of a breast cancer amplicon. Genome Res.

[49] Born M, Quintanilla-Fend L, Braselmann H, Reich U, Richter M, Hutzler P (2005). Simultaneous over-expression of the Her2/neu and PTK6 tyrosine kinases in archival invasive ductal breast carcinomas. J Pathol..

[50] Wang D, Zhu ZZ, Jiang H, Zhu J, Cong WM, Wen BJ (2015). Multiple genes identified as targets for 20q13.12-13.33 gain contributing to unfavorable clinical outcomes in patients with hepatocellular carcinoma. Hepatol Int..

[51] Miyoshi N, Ishii H, Mimori K, Nishida N, Tokuoka M, Akita H (2010). Abnormal expression of PFDN4 in colorectal cancer: a novel marker for prognosis. Ann Surg Oncol.

[52] Feng MY, Wang K, Shi QT, Yu XW, Geng JS (2009). Gene expression profiling in TWIST-depleted gastric cancer cells. Anat Rec.

[53] Hirota T, Takahashi A, Kubo M, Tsunoda T, Tomita K, Sakashita M (2012). Genome-wide association study identifies eight new susceptibility loci for atopic dermatitis in the Japanese population. Nat Genet.

[54] Guimaraes GS, Latini FR, Camacho CP, Maciel RM, Dias-Neto E, Cerutti JM (2006). Identification of candidates for tumor-specific alternative splicing in the thyroid. Genes Chromosomes Cancer.

[55] Santamaria I, Alvarez-Hernandez D, Jofre R, Polo JR, Menarguez J, Cannata-Andia JB (2005). Progression of secondary hyperparathyroidism involves deregulation of genes related to DNA and RNA stability. Kidney Int.

[56] Kwon OC, Lee EJ, Lee JY, Youn J, Kim TH, Hong S (2019). Prefoldin 5 and anti-prefoldin 5 antibodies as biomarkers for uveitis in ankylosing spondylitis. Front Immunol..

[57] Pells S, Koutsouraki E, Morfopoulou S, Valencia-Cadavid S, Tomlinson SR, Kalathur R (2015). Novel human embryonic stem cell regulators identified by conserved and distinct CpG island methylation state. PLoS ONE.

[58] Pi WF, Guo XJ, Xu XH, Ni PH, Xu WG (2007). Activation related genes of memory CD(4)(+) T cells in asthma patients. Zhonghua Jie He He Hu Xi Za Zhi..

[59] Deng YJ, Huang ZX, Zhou CJ, Wang JW, You Y, Song ZQ (2006). Gene profiling involved in immature CD4+ T lymphocyte responsible for systemic lupus erythematosus. Mol Immunol.

[60] Tsunoda T, Koh Y, Koizumi F, Tsukiyama S, Ueda H, Taguchi F (2003). Differential gene expression profiles and identification of the genes relevant to clinicopathologic factors in colorectal cancer selected by cDNA array method in combination with principal component analysis. Int J Oncol.

[61] Ariga H (2015). Common mechanisms of onset of cancer and neurodegenerative diseases. Biol Pharm Bull.

[62] Herberg JA, Beck S, Trowsdale J (1998). TAPASIN, DAXX, RGL2, HKE2 and four new genes (BING 1, 3 to 5) form a dense cluster at the centromeric end of the MHC. J Mol Biol.

[63] Igarashi S, Manabe A, Ohara A, Kumagai M, Saito T, Okimoto Y (2005). No advantage of dexamethasone over prednisolone for the outcome of standard- and intermediate-risk childhood acute lymphoblastic leukemia in the Tokyo Children’s Cancer Study Group L95-14 protocol. J Clin Oncol.

[64] Dehghan-Nayeri N, Rezaei-Tavirani M, Omrani MD, Gharehbaghian A, Goudarzi Pour K, Eshghi P (2017). Identification of potential predictive markers of dexamethasone resistance in childhood acute lymphoblastic leukemia. J Cell Commun Signal..

[65] Macaya-Sanz D, Chen JG, Kalluri UC, Muchero W, Tschaplinski TJ, Gunter LE (2017). Agronomic performance of *Populus deltoides* trees engineered for biofuel production. Biotechnol Biofuels.

[66] Lundin VF, Srayko M, Hyman AA, Leroux MR (2008). Efficient chaperone-mediated tubulin biogenesis is essential for cell division and cell migration in *C. elegans*. Dev Biol..

[67] Locascio A, Blazquez MA, Alabadi D (2013). Dynamic regulation of cortical microtubule organization through prefoldin-DELLA interaction. Curr Biol.

[68] Kadoyama K, Matsuura K, Takano M, Maekura K, Inoue Y, Matsuyama S (2019). Changes in the expression of prefoldin subunit 5 depending on synaptic plasticity in the mouse hippocampus. Neurosci Lett.

[69] Loke SY, Wong PT, Ong WY (2017). Global gene expression changes in the prefrontal cortex of rabbits with hypercholesterolemia and/or hypertension. Neurochem Int.

[70] Luder Ripoli F, Conradine Hammer S, Mohr A, Willenbrock S, Hewicker-Trautwein M, Brenig B (2016). Multiplex gene expression profiling of 16 target genes in neoplastic and non-neoplastic canine mammary tissues using branched-DNA assay. Int J Mol Sci.

[71] Luder Ripoli F, Mohr A, Conradine Hammer S, Willenbrock S, Hewicker-Trautwein M, Hennecke S (2016). A comparison of fresh frozen vs. formalin-fixed, paraffin-embedded specimens of canine mammary tumors via branched-DNA assay. Int J Mol Sci.

[72] Beck J, Hennecke S, Bornemann-Kolatzki K, Urnovitz HB, Neumann S, Strobel P (2013). Genome aberrations in canine mammary carcinomas and their detection in cell-free plasma DNA. PLoS ONE.

[73] Hennecke S, Beck J, Bornemann-Kolatzki K, Neumann S, Murua Escobar H, Nolte I (2015). Prevalence of the prefoldin subunit 5 gene deletion in canine mammary tumors. PLoS ONE.

[74] Yamane T, Shimizu T, Takahashi-Niki K, Takekoshi Y, Iguchi-Ariga SMM, Ariga H (2015). Deficiency of spermatogenesis and reduced expression of spermatogenesis-related genes in prefoldin 5-mutant mice. Biochem Biophys Rep..

[75] Henkel J, Du H, Yang P, Qyang Y, Kansra S, Ko M (2001). Bob1, a Gim5/MM-1/Pfd5 homolog, interacts with the MAP kinase kinase Byr1 to regulate sexual differentiation in the fission yeast, *Schizosaccharomyces pombe*. Differentiation..

[76] Gu Y, Deng Z, Paredez AR, DeBolt S, Wang ZY, Somerville C (2008). Prefoldin 6 is required for normal microtubule dynamics and organization in Arabidopsis. Proc Natl Acad Sci USA..

[77] Son HG, Seo K, Seo M, Park S, Ham S, An SWA (2018). Prefoldin 6 mediates longevity response from heat shock factor 1 to FOXO in *C. elegans*. Genes Dev.

[78] Cloutier P, Poitras C, Faubert D, Bouchard A, Blanchette M, Gauthier MS (2020). Upstream ORF-encoded ASDURF is a novel prefoldin-like subunit of the PAQosome. J Proteome Res.

[79] Zhang J, Liu L, Zhang X, Jin F, Chen J, Ji C (2006). Cloning and characterization of a novel human prefoldin and SPEC domain protein gene (PFD6L) from the fetal brain. Biochem Genet.

[80] Boulon S, Pradet-Balade B, Verheggen C, Molle D, Boireau S, Georgieva M (2010). HSP90 and its R2TP/Prefoldin-like cochaperone are involved in the cytoplasmic assembly of RNA polymerase II. Mol Cell.

[81] Gauthier MS, Cloutier P, Coulombe B (2018). Role of the PAQosome in regulating arrangement of protein quaternary structure in health and disease. Adv Exp Med Biol.

[82] Lichti J, Gallus C, Glasmacher E (2018). Immune responses—transcriptional and post-transcriptional networks pass the baton. Trends Biochem Sci.

[83] Cloutier P, Poitras C, Durand M, Hekmat O, Fiola-Masson E, Bouchard A (2017). R2TP/Prefoldin-like component RUVBL1/RUVBL2 directly interacts with ZNHIT2 to regulate assembly of U5 small nuclear ribonucleoprotein. Nat Commun..

[84] Horejsi Z, Takai H, Adelman CA, Collis SJ, Flynn H, Maslen S (2010). CK2 phospho-dependent binding of R2TP complex to TEL2 is essential for mTOR and SMG1 stability. Mol Cell.

[85] Chaves-Perez A, Thompson S, Djouder N (2018). Roles and functions of the unconventional prefoldin URI. Adv Exp Med Biol.

[86] Martinez-Fernandez V, Navarro F (2018). Rpb5, a subunit shared by eukaryotic RNA polymerases, cooperates with prefoldin-like Bud27/URI. AIMS Genet..

[87] Mita P, Savas JN, Djouder N, Yates JR, Ha S, Ruoff R (2011). Regulation of androgen receptor-mediated transcription by RPB5 binding protein URI/RMP. Mol Cell Biol.

[88] Chaves-Perez A, Yilmaz M, Perna C, de la Rosa S, Djouder N (2019). URI is required to maintain intestinal architecture during ionizing radiation. Science..

[89] Gu J, Liang Y, Qiao L, Lu Y, Hu X, Luo D (2015). URI expression in cervical cancer cells is associated with higher invasion capacity and resistance to cisplatin. Am J Cancer Res..

[90] Lipinski KA, Britschgi C, Schrader K, Christinat Y, Frischknecht L, Krek W (2016). Colorectal cancer cells display chaperone dependency for the unconventional prefoldin URI1. Oncotarget..

[91] Hu X, Zhang F, Luo D, Li N, Wang Q, Xu Z (2016). URI promotes gastric cancer cell motility, survival, and resistance to adriamycin in vitro. Am J Cancer Res..

[92] Miron-Garcia MC, Garrido-Godino AI, Garcia-Molinero V, Hernandez-Torres F, Rodriguez-Navarro S, Navarro F (2013). The prefoldin bud27 mediates the assembly of the eukaryotic RNA polymerases in an rpb5-dependent manner. PLoS Genet.

[93] Gstaiger M, Luke B, Hess D, Oakeley EJ, Wirbelauer C, Blondel M (2003). Control of nutrient-sensitive transcription programs by the unconventional prefoldin URI. Science.

[94] Martinez-Fernandez V, Garrido-Godino AI, Cuevas-Bermudez A, Navarro F (2018). The yeast prefoldin Bud27. Adv Exp Med Biol.

[95] Sun S, Tang Y, Lou X, Zhu L, Yang K, Zhang B (2007). UXT is a novel and essential cofactor in the NF-kappaB transcriptional enhanceosome. J Cell Biol.

[96] Taneja SS, Ha S, Swenson NK, Torra IP, Rome S, Walden PD (2004). ART-27, an androgen receptor coactivator regulated in prostate development and cancer. J Biol Chem.

[97] Enunlu I, Ozansoy M, Basak AN (2011). Alfa-class prefoldin protein UXT is a novel interacting partner of Amyotrophic Lateral Sclerosis 2 (Als2) protein. Biochem Biophys Res Commun.

[98] Zhao H, Wang Q, Zhang H, Liu Q, Du X, Richter M (2005). UXT is a novel centrosomal protein essential for cell viability. Mol Biol Cell.

[99] Satou A, Taira T, Iguchi-Ariga SM, Ariga H (2001). A novel transrepression pathway of c-Myc. Recruitment of a transcriptional corepressor complex to c-Myc by MM-1, a c-Myc-binding protein. J Biol Chem.

[100] Mori K, Maeda Y, Kitaura H, Taira T, Iguchi-Ariga SM, Ariga H (1998). MM-1, a novel c-Myc-associating protein that represses transcriptional activity of c-Myc. J Biol Chem.

[101] Hagio Y, Kimura Y, Taira T, Fujioka Y, Iguchi-Ariga SM, Ariga H (2006). Distinct localizations and repression activities of MM-1 isoforms toward c-Myc. J Cell Biochem.

[102] Farrell AS, Sears RC (2014). MYC degradation. Cold Spring Harb Perspect Med..

[103] Sakamuro D, Prendergast GC (1999). New Myc-interacting proteins: a second Myc network emerges. Oncogene.

[104] Luscher B, Larsson LG (1999). The basic region/helix-loop-helix/leucine zipper domain of Myc proto-oncoproteins: function and regulation. Oncogene.

[105] Fladvad M, Zhou K, Moshref A, Pursglove S, Safsten P, Sunnerhagen M (2005). N and C-terminal sub-regions in the c-Myc transactivation region and their joint role in creating versatility in folding and binding. J Mol Biol.

[106] Gaubatz S, Imhof A, Dosch R, Werner O, Mitchell P, Buettner R (1995). Transcriptional activation by Myc is under negative control by the transcription factor AP-2. EMBO J.

[107] Li H, Lee TH, Avraham H (2002). A novel tricomplex of BRCA1, Nmi, and c-Myc inhibits c-Myc-induced human telomerase reverse transcriptase gene (hTERT) promoter activity in breast cancer. J Biol Chem.

[108] Okano HJ, Park WY, Corradi JP, Darnell RB (1999). The cytoplasmic Purkinje onconeural antigen cdr2 down-regulates c-Myc function: implications for neuronal and tumor cell survival. Genes Dev.

[109] Taira T, Sawai M, Ikeda M, Tamai K, Iguchi-Ariga SM, Ariga H (1999). Cell cycle-dependent switch of up-and down-regulation of human hsp70 gene expression by interaction between c-Myc and CBF/NF-Y. J Biol Chem.

[110] Takayama M, Taira T, Iguchi-Ariga SM, Ariga H (2000). CDC6 interacts with c-Myc to inhibit E-box-dependent transcription by abrogating c-Myc/Max complex. FEBS Lett.

[111] Peukert K, Staller P, Schneider A, Carmichael G, Hanel F, Eilers M (1997). An alternative pathway for gene regulation by Myc. EMBO J.

[112] Niki T, Izumi S, Saegusa Y, Taira T, Takai T, Iguchi-Ariga SM (2000). MSSP promotes ras/myc cooperative cell transforming activity by binding to c-Myc. Genes Cells.

[113] Takayama MA, Taira T, Tamai K, Iguchi-Ariga SM, Ariga H (2000). ORC1 interacts with c-Myc to inhibit E-box-dependent transcription by abrogating c-Myc-SNF5/INI1 interaction. Genes Cells.

[114] Cheng SW, Davies KP, Yung E, Beltran RJ, Yu J, Kalpana GV (1999). c-MYC interacts with INI1/hSNF5 and requires the SWI/SNF complex for transactivation function. Nat Genet.

[115] Shrivastava A, Saleque S, Kalpana GV, Artandi S, Goff SP, Calame K (1993). Inhibition of transcriptional regulator Yin-Yang-1 by association with c-Myc. Science.

[116] Austen M, Cerni C, Luscher-Firzlaff JM, Luscher B (1998). YY1 can inhibit c-Myc function through a mechanism requiring DNA binding of YY1 but neither its transactivation domain nor direct interaction with c-Myc. Oncogene.

[117] Alexandrova N, Niklinski J, Bliskovsky V, Otterson GA, Blake M, Kaye FJ (1995). The N-terminal domain of c-Myc associates with alpha-tubulin and microtubules in vivo and in vitro. Mol Cell Biol.

[118] Taira T, Maeda J, Onishi T, Kitaura H, Yoshida S, Kato H (1998). AMY-1, a novel C-MYC binding protein that stimulates transcription activity of C-MYC. Genes Cells.

[119] Elliott K, Ge K, Du W, Prendergast GC (2000). The c-Myc-interacting adaptor protein Bin1 activates a caspase-independent cell death program. Oncogene.

[120] Kitaura H, Shinshi M, Uchikoshi Y, Ono T, Iguchi-Ariga SM, Ariga H (2000). Reciprocal regulation via protein-protein interaction between c-Myc and p21(cip1/waf1/sdi1) in DNA replication and transcription. J Biol Chem.

[121] Gu W, Bhatia K, Magrath IT, Dang CV, Dalla-Favera R (1994). Binding and suppression of the Myc transcriptional activation domain by p107. Science.

[122] Beijersbergen RL, Hijmans EM, Zhu L, Bernards R (1994). Interaction of c-Myc with the pRb-related protein p107 results in inhibition of c-Myc-mediated transactivation. EMBO J.

[123] Guo Q, Xie J, Dang CV, Liu ET, Bishop JM (1998). Identification of a large Myc-binding protein that contains RCC1-like repeats. Proc Natl Acad Sci USA.

[124] McEwan IJ, Dahlman-Wright K, Ford J, Wright AP (1996). Functional interaction of the c-Myc transactivation domain with the TATA binding protein: evidence for an induced fit model of transactivation domain folding. Biochemistry.

[125] McMahon SB, Van Buskirk HA, Dugan KA, Copeland TD, Cole MD (1998). The novel ATM-related protein TRRAP is an essential cofactor for the c-Myc and E2F oncoproteins. Cell.

[126] Kimura Y, Nagao A, Fujioka Y, Satou A, Taira T, Iguchi-Ariga SM (2007). MM-1 facilitates degradation of c-Myc by recruiting proteasome and a novel ubiquitin E3 ligase. Int J Oncol.

[127] Narita R, Kitaura H, Torii A, Tashiro E, Miyazawa M, Ariga H (2012). Rabring7 degrades c-Myc through complex formation with MM-1. PLoS ONE.

[128] Yoshida T, Kitaura H, Hagio Y, Sato T, Iguchi-Ariga SM, Ariga H (2008). Negative regulation of the Wnt signal by MM-1 through inhibiting expression of the wnt4 gene. Exp Cell Res.

[129] Ma HC, Lin TW, Li H, Iguchi-Ariga SM, Ariga H, Chuang YL (2008). Hepatitis C virus ARFP/F protein interacts with cellular MM-1 protein and enhances the gene trans-activation activity of c-Myc. J Biomed Sci.

[130] Satou A, Hagio Y, Taira T, Iguchi-Ariga SM, Ariga H (2004). Repression of the c-fms gene in fibroblast cells by c-Myc-MM-1-TIF1beta complex. FEBS Lett.

[131] Fujioka Y, Taira T, Maeda Y, Tanaka S, Nishihara H, Iguchi-Ariga SM (2001). MM-1, a c-Myc-binding protein, is a candidate for a tumor suppressor in leukemia/lymphoma and tongue cancer. J Biol Chem.

[132] Botchkarev VA, Flores ER (2014). p53/p63/p73 in the epidermis in health and disease. Cold Spring Harb Perspect Med..

[133] Watanabe K, Ozaki T, Nakagawa T, Miyazaki K, Takahashi M, Hosoda M (2002). Physical interaction of p73 with c-Myc and MM1, a c-Myc-binding protein, and modulation of the p73 function. J Biol Chem.

[134] Han A, Li J, Li Y, Wang Y, Bergholz J, Zhang Y (2016). p63alpha modulates c-Myc activity via direct interaction and regulation of MM1 protein stability. Oncotarget..

[135] Chen Y, Li Y, Peng Y, Zheng X, Fan S, Yi Y (2018). DeltaNp63alpha down-regulates c-Myc modulator MM1 via E3 ligase HERC3 in the regulation of cell senescence. Cell Death Differ.

